# Chronic Pseudo-Membranous Bronchitis

**Published:** 1887-01

**Authors:** H. A. Johnson

**Affiliations:** Emeritus Professor of Principles and Practice of Medicine in Chicago Medical College; 4, 16th street


### REPORT OF A CASE OF CHRONIC PSEUDO-MEMBRANOUS BRONCHITIS. *By* H. A. JOHNSON, M.D., LL.D., *Emeritus Professor of Principles and Practice of Medicine in Chicago Medical College.*

[Read before the Chicago Medical Society, December 20th, 1886.]

Pseudo-membranous bronchitis is rarely met with. In making this statement I exclude the persistence of a diphtheritic bronchitis and croupous pneumonia, in both of which diseases the expulsion of false membranes may occur. It is perhaps not always easy to make an absolutely correct differential diagnosis of these cases. This difficulty rests upon the fact that (1) membranous inflammation of the bronchii of an acute character, such as diphtheria, may become chronic. I have seen several such cases, but in all of them the acute stage had been well marked, and the chronic condition seemed to be only delayed convalescence. (2) Croupous pneumonia may certainly become chronic, but so far as my own experience enables me to judge, the membranous exudate disappears with the acute stage.

The literature of the subject is quite voluminous in titles, as may be seen by reference to the index catalogue of the library of the Surgeon-General's office, but the number of cases is small.

Among the cases reported in our own country, one by Dr. W. C. Glasgow, of St. Louis, in a paper read before the American Medical Association for the year 1879, is especially noticeable. In this article the author embodies the experience of several of the more prominent physicians of the United States.

Dr. Richardson, of New Orleans, "in a practice of nearly a third of a century" had "never encountered a case of plastic bronchitis."

Dr. Geddings, of Aiken, South Carolina, had "never met with a case." It should be remembered that Dr. Geddings had a very large experience in lung troubles.

Dr. F. R. Porcher, of Charleston, had seen one case.

Dr. T. G. Simons, of Charleston, had seen one case, and had known of three others in the practice of other physicians.

Dr. Jerome Cochran, of Mobile, says it is unknown in that section of the country.

Dr. James H. Hutchinson, of Philadelphia, had seen one case.

Dr. H. I. Bowditch, of Boston, had never seen a case.

Dr. R. H. Fitz, of Boston, had seen four specimens of casts. Does not seem to have seen the patients.

Dr. T. Parvin had seen no cases.

Dr. Geo. P. Andrews, of Detroit, had never seen or heard of a case in that region.

Dr. Roberts Bartholow had seen one well-marked case.

Dr. J. R. Leaming, of New York, had seen, in consultation, two cases.

Dr. Austin Flint, Sr., had seen three cases.

Dr. Gleitzman, of Ashville, North Carolina, had seen one case.

Dr. J. M. Da Costa, of Philadelphia, had specimens of casts from five cases; cannot say whether he had seen more cases.

Dr. Alfred Statle, of Philadelphia, sent report of one case.

Dr. P. E. Robinson, of St. Louis, reported one case.

Dr. Maxwell reports one case.

Dr. Samuel G. Armor, of Brooklyn, had seen "a few cases."

Dr. Frank Donaldson, of Baltimore, had seen one case.

Dr. Henry Gibbons, Sr., of San Francisco, had never seen a case during a practice of fifty years.

Dr. Charles Denison, of Colorado, had never seen or heard of a case in Colorado.

Dr. Baumgarten, of St. Louis, reports one case.

These facts collected by Dr. Glasgow in 1879, perhaps fairly represent the experience of the profession in America. I am, however, inclined to think that these meager statistics of the practice of some of the most active physicians and careful observers by no means give a correct estimate of the relative frequency of the affection. I imagine very many cases are never diagnosed, or if seen and recognized they are not recorded, and therefore lost sight of.

In the records of the literature upon this subject there are reports by L. H. Angel, CHICAGO MEDICAL JOURNAL, 1859, pp. 501 to 504.

J. S. Cohen, "Transactions of the Pathological Society," Philadelphia, 1876.

Austin Flint, Sr., "Medical Record," 1874.

J. H. Hutchinson, "Transactions of the Philadelphia Pathological Society," 1874.

A. L. Payne, *Stethescope and Va. Medical Gazette*, 1852.

J. C. Reeves, "Pathological Society," Philadelphia, 1859.

P. G. Robinson, *St. Louis Medical Journal*, 1878.

S. Rogers, "Transactions Medical Society of New York," 1866.

L. Smith, *Medical Record*, 1872.

T. H. Streets, *American Journal Medical Sciences*, 1880.

E. D. Worthington, *Canada Medical and Surgical Journal*, 1876.

These, in addition to the case reported by Dr. Glasgow, comprise all the titles I am able to find in the United States and Canada. They evidently include also some of the cases referred to in the correspondence reported by Dr. Glasgow and briefly summarized above.

In some of these cases it seems to me there was simply an acute or diphtheritic inflammation running its course in a few days and terminating in death, with such symptoms as are seen in the ordinary forms of diphtheritic inflammation.

Among foreign authorities the reports are also meager. Eichhorst, in the last German edition of his work on special pathology and therapeutics, finds only 100 cases on record.

The article in Ziemssen's Encyclopedia gives a very clear statement of what is known as to the etiology and pathology of the affection.

Among other writers Cheyne thinks old age predisposing; Valleix doubts this.

Gintrac says that the larger number of cases are observed in adult life. If we exclude the cases of diphtheria extending to the bronchii, this is true.

The male sex is predisposed to the affection according to most authorities. Enfeebled health from previous disease, poverty, fatigue, exposure, are among the most common causes noted. Of course all these are so many synonyms for ignorance. The cause remains to be discovered. It may be some local colony of parasites. The relation of this disease to the ordinary forms of membranous inflammation in some of which bacteria are believed to be a pathogenic factor suggests this, and perhaps makes it probable.

The relations to antecedent disease are by no means constant; neither diphtheria, nor simple bronchitis, nor pneumonia, except in rare instances, eventuate in chronic pseudo-membranous inflammation of the bronchial tubes.

Rugel says, "a special predisposition, or the influence of some special unknown agency is always essential in addition" to the hypothetical causes enumerated.

The pathology of the affection is better understood.

There is an exudate which coagulates upon the surface of the mucous membrane. This is often laminated by successive deposits. In the meshes of this coagulum a few leucocytes are found. The membrane proper is not necrosed, but continues to produce epithelium and the exudate is pushed off by the multiplication of this epithelium which in turn degenerates, becoming fatty and purulent. It seems also to be certain that while the mucous membranes do not become the seat of necrosis they do become the seat of morbid processes, possibly similar to that which in the endothelium of blood vessels determines the formation of a thrombus, and which in this case determines the formation of the plastic deposit.

The patient in this case is G. T. P., aged 37 years, a native of the eastern shores of the Adriatic. The family history on both sides is good. He enjoyed good health as a child and during his early manhood; at 17 had a suspicious sore, but apparently escaped any other manifestations of specific disease; was for several years a sailor, but abandoned that calling at the age of 25. Has been for some years keeping a saloon.

Eight years ago he gives a history of pneumonia involving the right lung; was six weeks ill. His general health from that time was good, till in March, 1884, when he "caught cold." At that time he was in bed ten days, had cough with expectoration, but does not know what was the character of the matter expectorated; had pain in the right side, locating it in the mammary region, this was not severe, but it continued more or less at intervals to the time of consultation. The cough and expectoration also continued during the spring and summer with, however, upon the whole a slow improvement till four days before first seen, when he thinks he caught cold, cough became more troublesome and he spat up once only a little blood. He consulted me on the 23d of August, 1884.

I found him a well-built man, 5 feet 7 inches in height, dark hair and eyes, weighing when well 147 pounds, but now evidently much reduced, 125 to 130 pounds. He stated that he had lost his weight since last winter; his appetite was poor; bowels torpid; urine normal in quantity but high in color; pulse 75, temperature 99.3, respiration 17 per minute, sleep fair, tongue coated. The cough and expectoration led him to fear phthisis, and the consultation was had with the expectation that there would be found evidence of that disease.

Upon inspection the chest was found to be noticeably flattened, but not more so on one side than on the other; over the right side and especially in the mammary region vocal fremitus exaggerated. Upon percussion there was found dullness over the whole right side, the left side normal.

Ausculation revealed bronchial expiration over superior portion of right side, front and back. Left side normal. Cardiac sounds normal. The diagnosis then entered in the case book was a "pneumonia not completely resolved, with bronchitis." He was placed upon tonics, syrup hypophosphites with hydrobromic acid.

September 9th. Seventeen days later, he came again, and in every respect seemed to be better. No physical examination was made, but he was ordered to continue the medicine.

September 16th. After quite a severe coughing fit and the expulsion of a mass of what was found to be a cast of a large bronchus, he spat blood. The hemorrhage persisted, and he was ordered extract ergot in capsules, and to continue the syrup hypophosphites and acid hydrobromic. The diagnosis was corrected so as to read "chronic pseudo-membranous bronchitis."

September 23rd. Bleeding continued two days after last visit, none since. Has had a great deal of pain in the interscapular region, not more on one side than the other. At times very tender to the touch at right of the eighth dorsal vertebra. This he describes as a "soreness." Has expectorated thin pieces of membrane since last consultation.

Physical examination. Find no dullness over the right side, or as the record says, "no noticeable difference in the percussion noted on the two sides." This was one month after the first examination, when there was dullness over the whole of the right side. The breath sounds over the right side feeble, in every other respect normal. Pulse 68, temperature 98.6.

October 2nd. Had been doing well until yesterday, when he again coughed up a large cast of a bronchus. (I may remark that all of these which I saw were probably from the first and second size tubes, and were from two to four inches in length.) After this there came what he describes as pus streaked with blood, but the hemorrhage not copious. The ergot had been stopped; he thinks he was better while taking it and asks to be permitted to return to it.

October 17th. The casts continue to be coughed up; microscopically they consist of coagulated plasma with a few leucocytes. Since the last date, October 2nd, he had been taking balsam copaiba and oleores. cubeb. with the ergot. I was under the impression that the copaiba had increased the plasticity of the exudate. Keeping in mind the specific history in his early life I thought possibly that there might be some lingering impression still. I therefore put him on pot. iodid. 0.50 t. i. d.

October 25th. Casts continue almost daily; continue pot. iodid. and add hydr. prot. iodid. 0.01 t. i. d.

November 5th, his wife comes to the office, says that he has thrown off a large number of casts, and each is followed by copious hemorrhage. Has continued to take the ergot, and is now a little better but weak; continue pot. iodid. and hydr. prot. iodid. and in addition ℞ elix. calisayæ 450 and acid. sulphur-arom. 50.00 grams M. and take a dessertspoonful in water three times daily.

November 21st, patient comes himself. Has been better since last date. Has had no hemorrhage, or but little. Still a few casts, appetite has improved under the tonic. Bowels regular and sleep good.

December 26th, has been doing well till recently, but is now evidently losing in weight and strength. Hemorrhages from chest and occasionally from nose. Appetite poor. Bowels regular, or occasionally diarrhœa. This, however, does not persist. Has lancinating pains in the abdomen, more in the epigastric region. Coughs up very few casts and these very thin and delicate. Has taken now the hydr. prot. iodid. since the 25th of October, 0.01 three times daily, and a part of the time 0.50 pot. iodid. He has also taken, according to the amount of hemorrhage, ergot at his own discretion. Stop both ergot and hydr. prot. iodid. and take syr. fe. iodid. c. c. 1.00 t. i. d.

January 9th, 1885, has been feeling better for the last two weeks. Appetite fair, bowels regular, no more pains in bowels since change in medicine, cough less, expectoration mucous, occasionally tinged with blood. No free bleeding and no casts. Has some pain in chest, bilateral, and not marked at any one place. Pulse 78, temperature 98, respiration normal.

During the last week in January his wife came, said that he was still coughing a little and that the expectoration was streaked with blood. I directed an emulsion of oleum terebinth, each dose containing 0.50 of the oil, three times daily, and to omit the ferri iodid.

February 4th, he was visited at his home. He has expectorated no casts since December 26th, 1884, but continues to cough sputum streaked with blood, and occasionally very slight epistaxis. Is still taking the turpentine; thinks the cough is looser than when taking the iron. During the past week has had a good deal of pain, intermittent in character, in the lower half of the right chest; has been in bed for last three days because of this pain.

Upon examination find the motions of the lower right side restricted; on percussion, dullness over the lower third of right lung, line of dullness seems to change with change of position; breath sounds indistinct, voice sounds exaggerated. Friction sounds distinctly heard over anterior portion of chest when patient lying on back; less so when patient is sitting up. Diagnosed, pleurisy, question of effusion doubtful; a hypodermic needle was introduced with negative results. Chest was ordered to be painted with iodine. The turpentine was continued.

Dr. Frank S. Johnson, to whom I am indebted for the larger portion of these notes, had made this visit, and on his return from the patient, in the extreme northwestern part of the city, became seriously ill. I was unable to look after the patient, and I asked my friend, Dr. S. D. Jacobson, to take charge of the case. This was, I think, on the 5th of February, 1885.

I beg to add extracts from a letter from Dr. Jacobson, giving in a general way the further treatment of the case:

"As to my ideas about the therapeutics of this case I can be short. I am not troubled with an *embarras de richesse*, but rather find my excuse in the old saw, *simplex sigillum veri*.

"The case was to me one of great interest, having never seen a similar one in twenty-five years of practice, and finding little or no mention of such cases in the books at my disposal. True, I have had one case of bronchial croup, which terminated fatally in a couple of days (a man about forty-eight years old). But your case had already been under your care and observation for several months before I saw him.

During the earlier months of my attendance he was about the same as when you saw him, intensely harassing cough with dyspnœa until relieved by the expulsion of greater or smaller masses of bronchial casts, which relief was generally paid for by severe hemorrhages, which told on the little strength he possessed before, so that he not only dwindled down to a skeleton-like appearance, but when able to sit up his legs would not support him, and his hands grew so weak that he could not for some time lift the spoon to his mouth. During the summer of 1885 he improved some, but the fall and winter reduced him below his former level.

"Having no authorities to guide me in the treatment of such a rare case, I applied the general principles to the best of my abilities. I had two indications before me, (1) *indicatio symptomatica*, and (2) *indicatio morbi.* As to the first class, I had in view the cough, dyspnœa, hemorrhages, weakening of all the organs and functions. Those I tried to meet by the exhibition of *solventia*, *expectorantia*, *narcotica*, *styptica et roborantia.* As to the *indicatio morbi* I was more in the dark, knowing almost nothing about the pathology or etiology of this disease. But I reasoned like this: Since our pathology seems to drift more and more into bacteriology, it is but just that our therapeutics follow suit and be more in the nature of bactericides and antiseptics. In this light I wish you to judge my prescriptions containing such poisons as arsenic, iodine, bi-chloride hydrarg. and iodoform, which appear in many of them.

"I must confess that my success was a great deal more than I dared to hope for, and though I firmly believe in the *vis medicatrix naturæ*, I also believe that a physician can be, and should be, in the words of Lord Bacon, '*medicus naturæ minister et interpres.*'"

During the summer of 1886, and again within the last few weeks, I have seen the patient, and find him perfectly recovreed.

4, 16th street.

